# What Is the Proper Field of the American Veterinary Medical Association?

**Published:** 1902-02

**Authors:** 


### WHAT IS THE PROPER FIELD OF THE AMERICAN VETERINARY MEDICAL ASSOCIATION?

In answering this question, let us recall that there are at this time a large number of very active local veterinary societies, and in each State in which the veterinary sciences have attained any degree of prominence there is a more or less efficient State Veterinary Society.

These organizations hold monthly, quarterly, semi-annual, or annual meetings that bring together the best veterinarians within their respective fields, and, by invitation, frequently obtain the benefit of counsel with distinguished veterinarians from distant places. They provide for the reading of papers and for demonstrations on the chief topics of interest to the general practitioner.

Veterinarians have an opportunity in these meetings to discuss with their colleagues the difficult and unusual cases that they may have met, and to obtain the benefit of counsel and advice. If a matter of legislation is under consideration it is discussed and action is taken in regard to it. At these meetings positive information is imparted and the policy of the profession is established.

The local and State veterinary societies have an enormous field of usefulness. The *esprit de corps* of the profession of a community depends largely upon the activity and standing of these societies. The States that have good laws governing the practice of veterinary medicine and the repression of disease have good State veterinary societies back of these laws. It is of the highest importance—it is absolutely essential—to the welfare of the veterinary profession in this country that the local and State veterinary societies shall be sustained and strengthened.

If these societies fulfil all of the requirements of the veterinary profession there is no need of other societies for veterinarians. But, in addition to the general and local questions that have been mentioned, there are other higher questions that affect the country and profession as a whole. There are certain questions that are distinctly national, just as there are certain questions for local boards of health and questions for State live-stock sanitary boards, and, at the same time, a distinct field of usefulness for the United States Bureau of Animal Industry.

If the United States Bureau of Animal Industry were to attempt to do the work of local and State sanitary organizations the work of these local organizations would be interfered with, the organizations themselves would be weakened, and the proper work of the national bureau would be crowded and impeded.

In the same way the American Veterinary Medical Association has its distinct field. By infringing upon the proper fields of usefulness of local and State Veterinary Societies it weakens these local organizations, it excludes its own proper work, and weakens itself.

The American Veterinary Medical Association is the Supreme Court, it is the Senate, it is the Carnegie Institution of the veterinary profession.

Why should petty police justice cases be tried in the Supreme Court or village ordinances be thrashed out in the Senate, or why should grammar-school instruction be given in the highest university in the land?

For the sake of the local and State veterinary societies, for the sake of the American Veterinary Medical Association, we hope that our national organization will "find itself."

